# Thermal Stress Promotes Host Mitochondrial Degradation in Symbiotic Cnidarians: Are the Batteries of the Reef Going to Run Out?

**DOI:** 10.1371/journal.pone.0039024

**Published:** 2012-07-16

**Authors:** Simon R. Dunn, Mathieu Pernice, Kathryn Green, Ove Hoegh-Guldberg, Sophie G. Dove

**Affiliations:** 1 ARC Centre of Excellence for Coral Reef Studies, School of Biological Sciences, University of Queensland, Brisbane, Australia; 2 Laboratory for Biological Geochemistry, School of Architecture, Civil and Environmental Engineering, Ecole Polytechnique Fédérale de Lausanne, Lausanne, Switzerland; 3 Centre for Microscopy and Microanalysis, University of Queensland, Brisbane, Australia; 4 Global Change Institute, University of Queensland, Brisbane, Australia; Texas A&M University, United States of America

## Abstract

The symbiotic relationship between cnidarians and their dinoflagellate symbionts, *Symbiodinium* spp, which underpins the formation of tropical coral reefs, can be destabilized by rapid changes to environmental conditions. Although some studies have concluded that a breakdown in the symbiosis begins with increased reactive oxygen species (ROS) generation within the symbiont due to a decoupling of photosynthesis, others have reported the release of viable symbionts via a variety of host cell derived mechanisms. We explored an alternative model focused upon changes in host cnidarian mitochondrial integrity in response to thermal stress. Mitochondria are often likened to being batteries of the cell, providing energy in the form of ATP, and controlling cellular pathway activation and ROS generation. The overall morphology of host mitochondria was compared to that of associated symbionts under an experimental thermal stress using confocal and electron microscopy. The results demonstrate that hyperthermic stress induces the degradation of cnidarian host mitochondria that is independent of symbiont cellular deterioration. The potential sites of host mitochondrial disruption were also assessed by measuring changes in the expression of genes associated with electron transport and ATP synthesis using quantitative RT-PCR. The primary site of degradation appeared to be downstream of complex III of the electron transport chain with a significant reduction in host cytochrome *c* and ATP synthase expression. The consequences of reduced expression could limit the capacity of the host to mitigate ROS generation and maintain both organelle integrity and cellular energy supplies. The disruption of host mitochondria, cellular homeostasis, and subsequent cell death irrespective of symbiont integrity highlights the importance of the host response to thermal stress and in symbiosis dysfunction that has substantial implications for understanding how coral reefs will survive in the face of climate change.

## Introduction

The symbiotic relationship between cnidarians, in particular scleractinian corals, and their dinoflagellate symbionts, *Symbiodinium* spp, underpins the foundation and formation of tropical coral reefs [1]. However, under rapid or extreme changes in environmental conditions, the symbiosis becomes unstable and can break down, resulting in the loss or *in-situ* degradation of the symbionts signified by a pale or bleached appearance of the cnidarian host. The re-occurrence of bleaching events can subsequently lead to partial or complete colony mortality [2], [3], which in turn, may have important ramifications for the productivity and growth of reefs on a global scale [4], [5]. Environmental stresses, such as high temperature and light (PAR and UV), as well as a number of other stressors linked to anthropogenic activity, can interact and trigger bleaching [6]. Despite growing knowledge about biology and ecology of mass coral bleaching, the cellular mechanisms that initiate the disruption of the symbiotic relationship are still being elucidated. In this regard, the breakdown of the symbiosis involves a range of integrated processes including immunity, non-self-recognition, cellular communication, oxidative stress, and cell death pathways [3]. Much of the current consensus is that the initiation of the bleaching response stems from the decoupling of photosynthesis at maximum functional rates resulting in damage of the photosystem II apparatus and a subsequent production of reactive oxygen species (ROS). The corresponding membrane and protein damage from excess ROS production in both the coral host and the symbiont and inevitably a breakdown in carbon fixation, ATP and NADH production [3]. However, recent perspectives on photoinhibition and photoprotection [7] have, argued against runaway photosynthetic ROS generation. Suggesting instead, that small increases in ROS production within the photosystems inhibit protein synthesis and hence both D1 repair of the reaction centers [8] and de novo antennal protein synthesis [9]. Such actions may lead to a photo-protective closure of the reaction centers [10], and overtime, to the dismantlement of the light harvesting antennae under heat stress [11]. If these recent perspectives are correct, then an initial production of ROS could prevent further ROS generation by closing down electron transport, light energy harvesting and consequentially, reduced carbon fixation. This position is entirely consistent with multiple different primary site of damage within the photosynthetic apparatus as recently observed by Buxton *et al.* [12] in different corals and algal clades. There are clearly multiple routes of degeneration, which can occur within the dinoflagellate symbiont.

Whilst the consensus of opinion regarding initiation of the breakdown of symbiosis has centred on the dysfunction of the photosynthesis, the mitochondria as an alternative source of potential harmful ROS in both the dinoflagellate and host cnidarian has been neglected. Earlier work by Nii and Muscatine [13] and Dykens *et al.* [14] highlighted the role of host mitochondria as both a source of ROS and antioxidant enzyme activity during a stress response. However, the correlation of such activity to a breakdown in symbiosis and cellular homeostasis during thermal stress was not explored further. In addition, the important role of the mitochondrial machinery in controlling cellular processes such as ATP production, oxidative phosphorylation and cell death pathways highlights the mitochondria as a potential pivotal component in the breakdown of symbiosis and onset of the bleaching process.

Of the proteins associated with mitochondrial function, cytochrome *c* (cyt*c*) is potentially one of the most important, with multiple roles in oxidative phosphorylation, electron transport, mitigation of oxidative stress and initiation of apoptotic programmed cell death. Cyt*c* is a water soluble, single chain haemoprotein that is located on the outside surface of the inner membrane of eukaryotic mitochondria, but is encoded by a nuclear gene [15]. This highly conserved protein facilitates electron transport by ‘shuttling’ between complex III (cyt*c* reductase) and complex IV (cytochrome oxidase) and is integral to the electron transport chain that results in ATP production required for the cells’ energy and survival [15]. Cyt*c* plays an important role in the removal of potentially damaging free radicals that are generated in the mitochondria by accepting electrons from superoxide radicals and delivering them to complex IV [16], and can contribute to the formation of antioxidant enzymes, such as peroxidase [15]. The release of cyt*c* from the mitochondria can initiate the highly conserved, intrinsically mediated apoptosis signalling cascade leading to cell disposal [17], [18], [19].

Amongst the cellular mechanisms that are known to be active in the release and degradation of the symbiotic dinoflagellates during a stress response are necrosis and the programmed cell death pathways, autophagy and apoptosis [20]. The apoptotic signalling cascade consists of three phases of activity: induction, execution and removal of cells from the host tissues. The induction may be mediated through either extrinsically activated receptors, such as FADD, procaspase 8 and 10 [21], [22] or intrinsically through the mitochondrial release of cyt*c* [17]. Downstream of the induction is the execution phase mediated by a cascading suite of, proteolytic enzymes known as caspases, [23], which translocate from the cytosol to the nucleus and activate endonucleases, DNA cleavage and cell disposal through compartmentalisation and phagocytosis of “apoptotic bodies” by surrounding cells [24]. One link between the induction and execution phases is characterised by the release of cyt*c* from the mitochondria into the cytosol, which acts in combination with ATP and *Apaf*1 to form the apoptosome and transition into the execution phase [19], [21]. However, if ATP is reduced, the controlled progression through to the execution phase may be disrupted, defaulting to an uncontrolled necrotic outcome [25].

The release of cyt*c* from the mitochondria can occur through rupture of the outer organelle membrane or selectively controlled by membrane pore formation. Different hypotheses relating to pore formation have been put forward, such as a mitochondrial permeability membrane transition pore (MPMT) or the voltage-dependent anion channel (VDAC) and associated adenine nucleotide translocator protein complex (ANT) [19], [26]. The formation of inner membrane pores may also alter the osmotic potential of the mitochondrial matrix and inter-membrane space, resulting in outer membrane rupture [19]. Cyt*c* may be released independently through activity of cyclophilin D, although this has also been attributed to the onset of cell nitric oxide-induced necrosis [27]. The upregulation of nitric oxide (NO) signalling has been previously shown to be active during the thermal stress-induced breakdown of the cnidarian-dinoflagellate symbiosis [28], [29]. One of the most recognised controls over pore formation is the interaction of pro and anti apoptotic members of the Bcl-2 family of proteins. Over-expression of pro-apoptotic members such as Bax can induce pore formation, whereas higher expression of anti-apoptotic Bcl-2 can prevent the release of cyt*c* by blocking the action of pro-apoptotic members [19], [22], [30]. Bcl-2 also has the capacity to act as an antioxidant to reduce ROS activity [19], [31]. The activity of ROS in different areas of the mitochondrial membranes releases cyt*c* directly through the peroxidation of cyt*c* binding complex or indirectly via the breakdown of membranes through lipid peroxidation [19], [31]. The interaction of pro and anti-apoptotic Bcl-2 family members has already been shown to be an important regulatory process during the onset of coral bleaching [32], [33].

In general, a high metabolic activity of cells and tissues requires a greater supply of oxygen and ATP, and so a greater number of mitochondria are required to meet the demand [34]. Cnidarian host muscular tissues and associated cells with a high surface area of cilia (including the gastrodermal cell layer that harbours the dinoflagellate symbionts) exhibit a higher demand for ATP and thus require increased numbers of mitochondria [35]. In addition, a high turnover of ATP from abundant mitochondria may be essential to the function of the calicoblastic tissue layer in hermatypic corals during the process of skeletogenesis [36]. Cells with a higher proportion of mitochondria may also have a proportionally higher susceptibility to ROS generation than cells with a lower metabolic demand. However, the most influential factor upon redox regulation within symbiotic cnidarians, is the photosynthetic dinoflagellate with its own chloroplasts, mitochondria and associated ROS generation potential within the cells of the host gastrodermis. Consequently, the symbiotic gastrodermal cell consortium is subjected to significant changes in redox state during a diurnal period, whereby changes in light, photosynthesis, symbiont and host respiration can generate hypoxic and hyperoxic conditions and subsequent changes in metabolic activity and ROS generation potential [3], [37], [38]. The regulation of the redox state to maintain cellular homeostasis in the response to the increased potential for ROS production can therefore place additional demands upon the host cell mitochondrial function associated with the symbiosis in comparison to other host cells.

The generation of excessive ROS and resulting effects upon cnidarian-dinoflagellate symbiosis are well documented during environmental stress-induced bleaching [39]. Elevated temperature and light (PAR and UV) in particular, have been linked to the generation of ROS [40], [41], such as O_2_ **.-** and OH**.** [14], [42] and NO [28], resulting in damage to both the photosynthetic apparatus [11], [43] and membrane lipids [44] of the symbiont and host. Whereas the different cell death mechanisms that have been shown to be active during bleaching [20], [45], are associated with ROS production, the pivotal link between the onset of host cell death, disruption of host cell organelles, ROS generation, energy limitation and specific molecular triggers has not been described. This study used the non-calcifying symbiotic cnidarian model *Aiptasia sp.*to focus on the integrity of host mitochondria when challenged with thermal stress and to explore any associated link between the onset of host mitochondrial and symbiont degradation. By investigating potential initial sites of degradation within the symbiotic cnidarian host mitochondria in response to thermal stress, this study aims to provide further insight into the molecular components and cellular pathways responsible for a host driven breakdown of this symbiosis.

## Results

### Differential Morphology of Host Cell Mitochondria in Heat Stressed Versus Control Conditions

**Figure 1:**
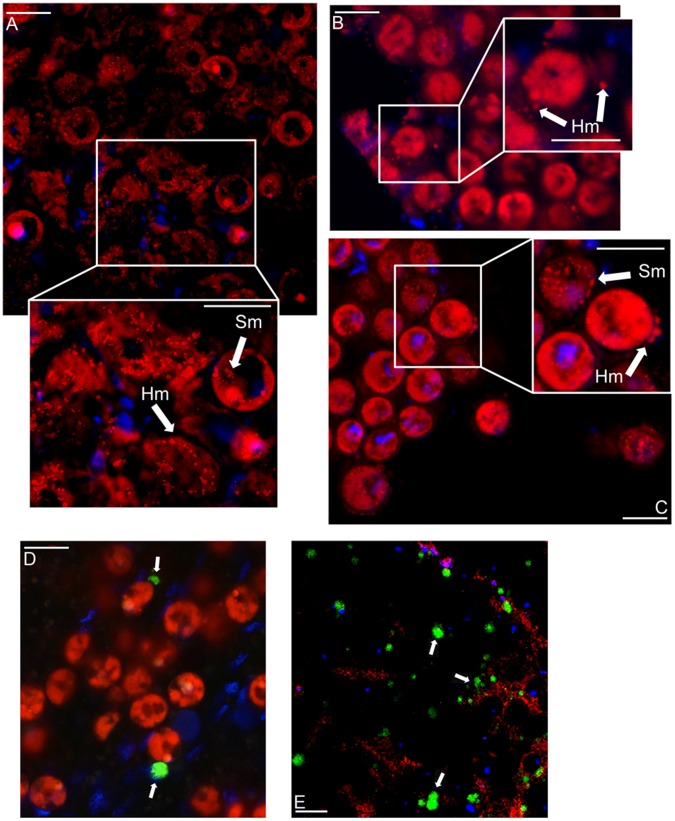
Laser Confocal Microscopy images of *Aiptasia pulchella* host (Hm) and dinoflagellate symbiont (Sm) mitochondria stained red with mitotracker ™ fluoroprobe. A) Control; B and C) Thermal stress treated individuals with highlighted expanded windows; D) Host cell caspase activation in thermal stressed individual anemones shown by rhodamine 110 green fluorescence (arrow) and degraded tissue sections induced by 0.5% colchicine incubation as a positive control (E). Scale Bar: 10 µm.

The mitotracker staining of the cryostat-prepared tissue sections identified the location of both host and dinoflagellate mitochondria under low magnification as small punctate concentrations of dye, in contrast to the background red-wash autofluorescence of the dinoflagellate chlorophyll (Figure 1A). A proportion of host gastrodermal cell mitochondria within anemones exposed to thermal stress appeared to aggregate and display a swollen and distended appearance, which were noticeably absent in the control host cells (Figure 1B and C). In contrast to the degradation of host mitochondria in response to the heat treatment, there was no direct evidence from the confocal microscopy data analysis to suggest that dinoflagellate mitochondria were deteriorating within the same treatment (Figure 1B and C). In addition to mitochondrial morphological changes, there was host caspase activation, implying execution of apoptosis in heat-treated anemones (Figure 1D) albeit at lower levels than chemically- induced positive controls (Figure 1E).

**Figure 2:**
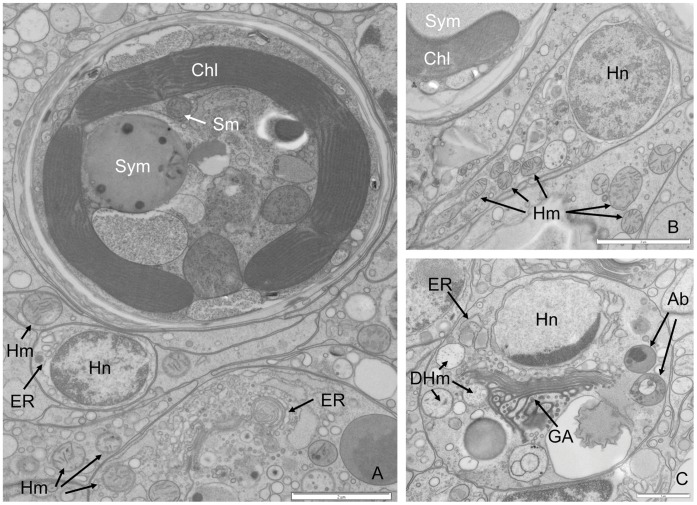
Electron micrograph showing host anemone cells and symbiont and specifically mitochondria from control untreated conditions. (A) Host cells and Symbiont (B) Host cell (C) Early apoptotic host cell defined by degraded mitochondria adjacent to the Golgi Body, a number of membrane- bound bodies containing cell components within the cytosol, nuclear chromatin crescentic cap condensation and perforations of the nuclear envelope. Key: Sym =  Symbiont, Sm  = symbiont mitochondria, Chl  =  Chloroplast, Hn  =  Host nucleus, Hm  =  Host mitochondria, GA  =  Golgi Apparatus, ER  =  Endoplasmic Reticulum DHm  =  Degraded Host mitochondria, Ab  =  Apoptotic body. (Scale Bars as shown).

**Figure 3:**
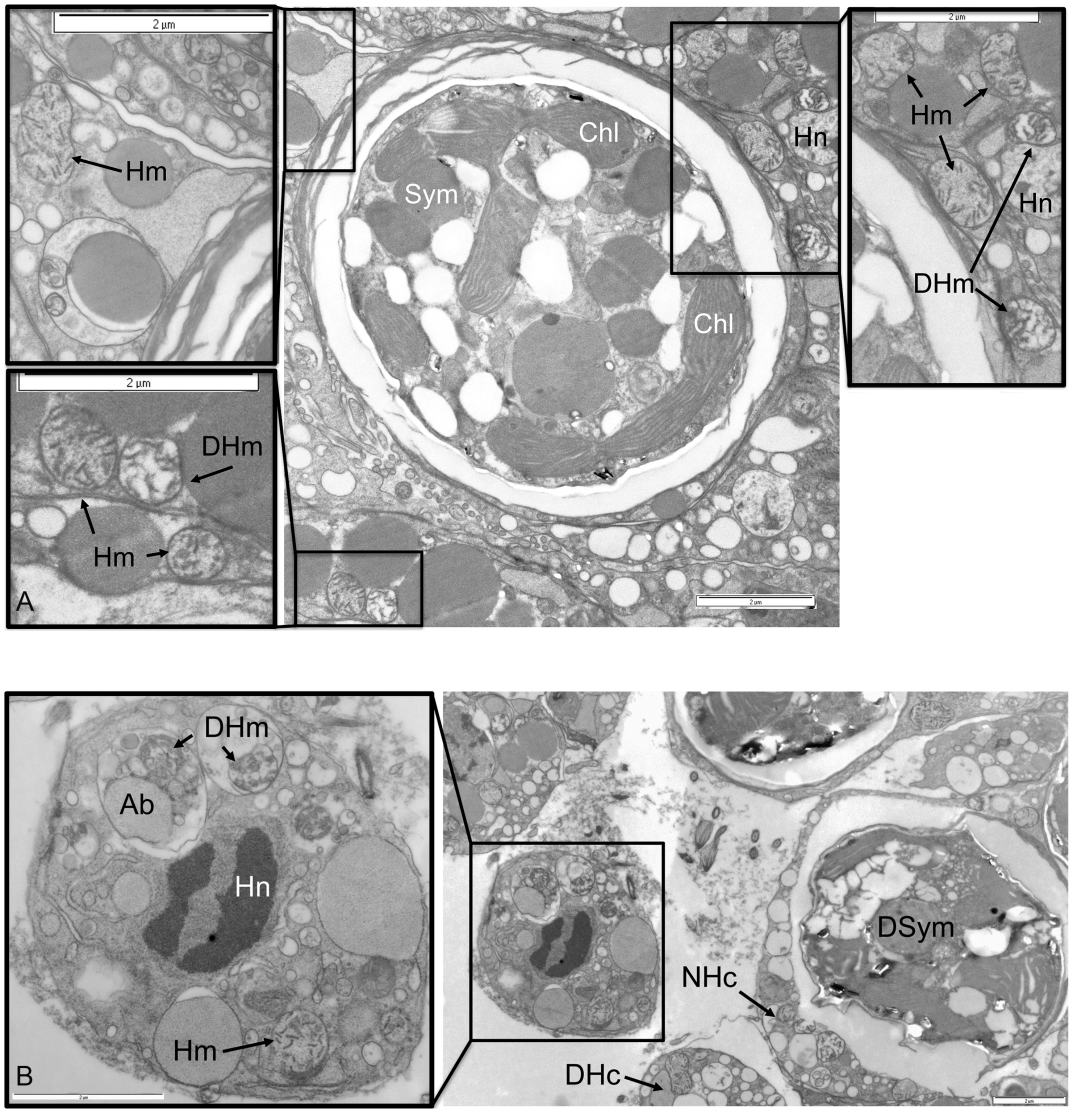
Electron micrograph showing host anemone cells with symbiont and mitochondria from heat treatment. (A) Host cells and symbiont, (B) Detachment of host apoptotic cell adjacent to ruptured necrotic cell. Key: Sym  =  Symbiont, DSym  =  Degraded Symbiont, Chl  =  Chloroplast, Hn  =  Host nucleus, Hm  =  Host mitochondria, GA  = Golgi Apparatus, ER  =  Endoplasmic Reticulum, DHm  =  Degraded Host mitochondria, Ab  =  Apoptotic body, NHc  =  Necrotic host cell, DHc  =  Detached Host cell (Scale Bars as shown).

**Figure 4:**
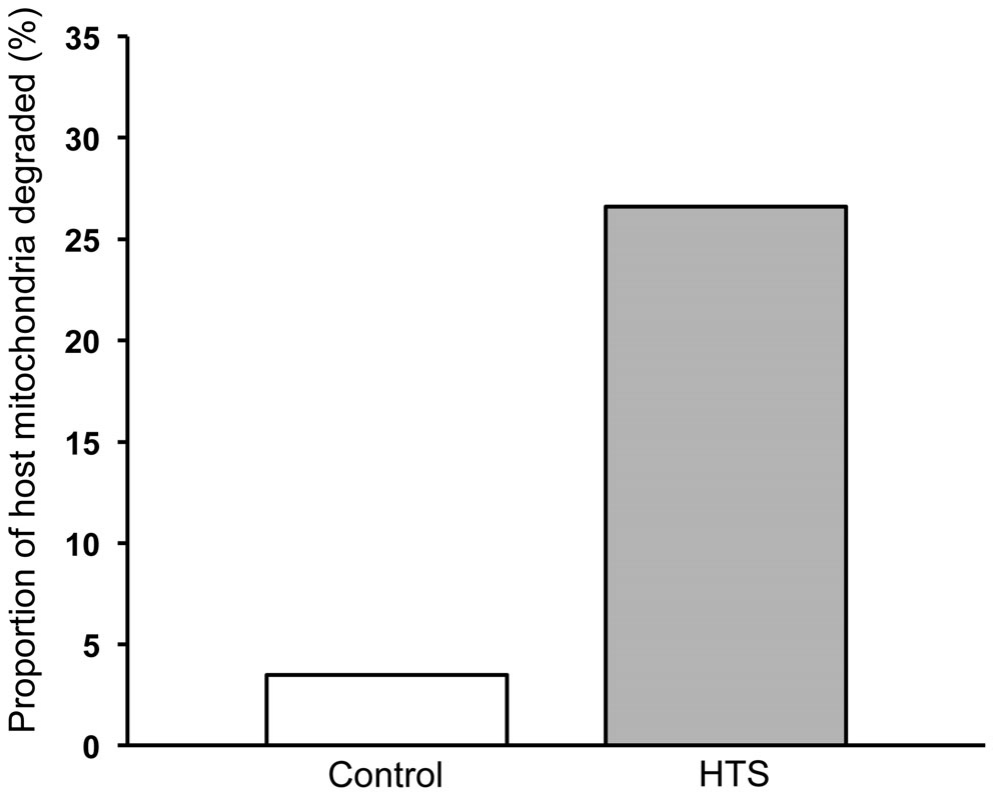
Proportion of host mitochondria displaying degraded morphology within control and hyperthermic stress treatment expressed as percentage of total observed.

**Figure 5:**
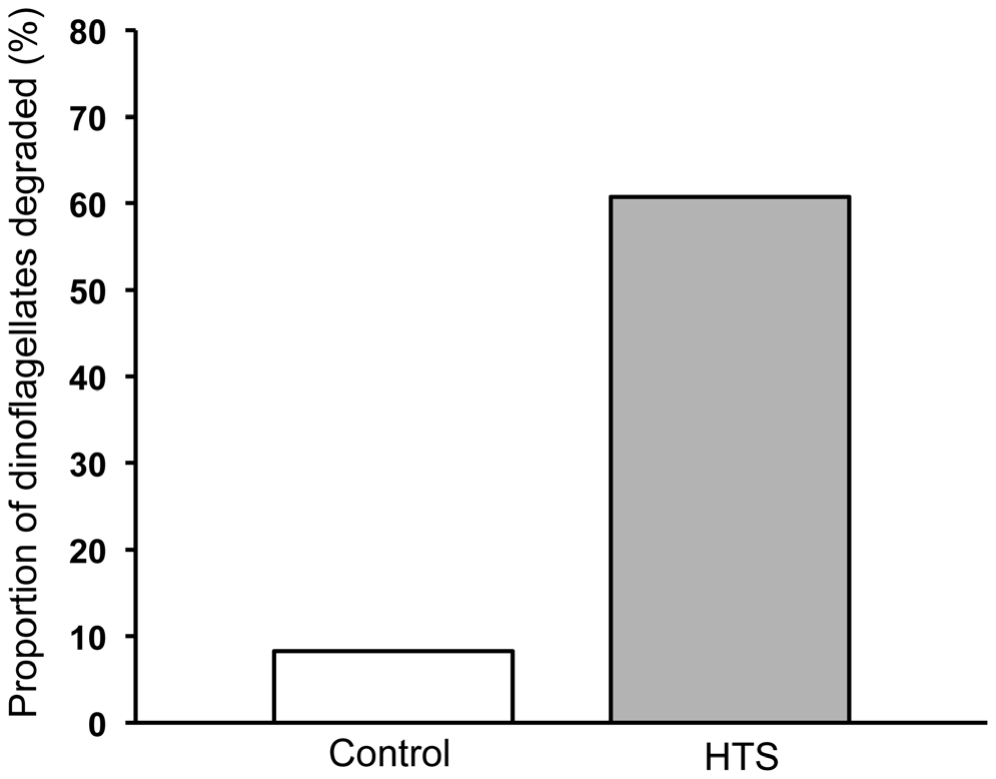
Proportion of symbiotic dinoflagellates displaying degraded morphology within control and hyperthermic stress treatment expressed as percentage of total observed.

**Figure 6:**
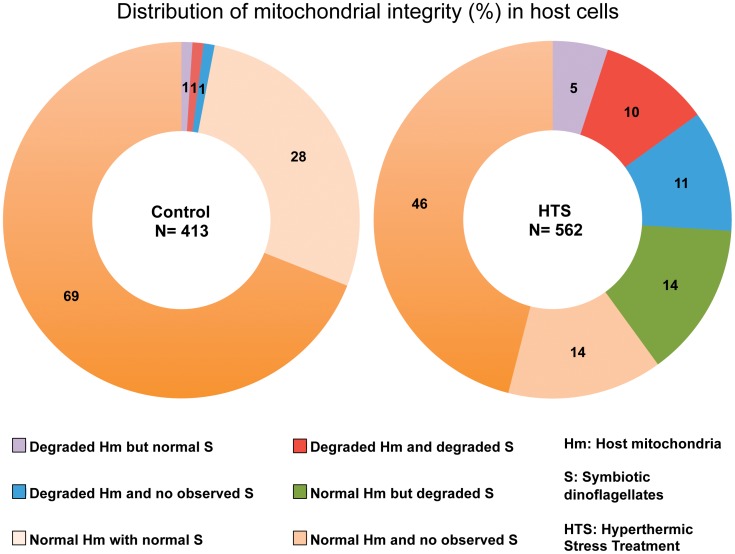
Distribution of host mitochondria (Hm) with either normal or degraded integrity and association with normal or degraded symbiotic dinoflagellates (S) in anemones subjected to Hyperthermic Stress Treatment (HTS) compared with control.

The detailed analysis of the ultrastructural integrity of host mitochondria within anemones from the control conditions demonstrated that the majority of organelles were uniform in appearance and their structure remained unaltered (Figure 2A and B). However, control animal tissues did include a small number of cells that did show signs of degradation and apoptotic host cell death (Figure 2C). The host mitochondria from the heat-treated animal tissues appeared to display either a healthy normal appearance or differential levels of degradation ranging from swollen and distended with separation of cristae and organelle membranes, which often appeared ‘ragged’, through to total organelle destruction [46], [47] (Figure 3A and B). In comparison with controls, the amount of degraded host mitochondria in heat-treated anemone cells was significantly higher (χ^2^_(1)_ = 96.32, P>0.0001) (Figure 4). In addition, there was a significant increase in the degradation of symbiotic dinoflagellates within heat-treated anemone host tissues (χ^2^_(1)_ = 52.59, P>0.0001) (Figure 5). Host apoptotic and necrotic cell death, host cell detachment and *in situ* dinoflagellate degradation were all observed within heat-treated anemone tissues (Figure 3B). The association of degraded host mitochondria with degraded symbiont dinoflagellates appeared to be disparate within some cells, with host mitochondria retaining integrity whilst dinoflagellates appeared to be degrading (not present in controls, but 14% in heat treated) and likewise, whereby healthy dinoflagellates were within host cells with degraded mitochondria (1% in controls compared to 5% in heat-treated) (Figure 6). Equally, some cells contained both degraded host and symbiont organelles (1% in controls compared to 10% in heat-treated) and other cells retained the integrity in both (69% in controls compared to 48% in heat-treated) (Figure 6). The difference between the distribution of both normal and degraded mitochondria and dinoflagellates in control and heat-treated anemones were highly significant in all 6 scenarios (χ^2^_(1)_ values not shown for each comparison, but all P>0.0001; Figure 6). The distribution of measured changes in host mitochondrial degradation with respect to the presence or absence of normal or degraded symbionts indicated that host mitochondrial degradation under temperature stress was independent of the symbiont integrity (Figure 6).

### Changes in Mitochondrial Gene Expression Associated with Electron Transport, ROS Generation and ATP Synthesis

**Figure 7:**
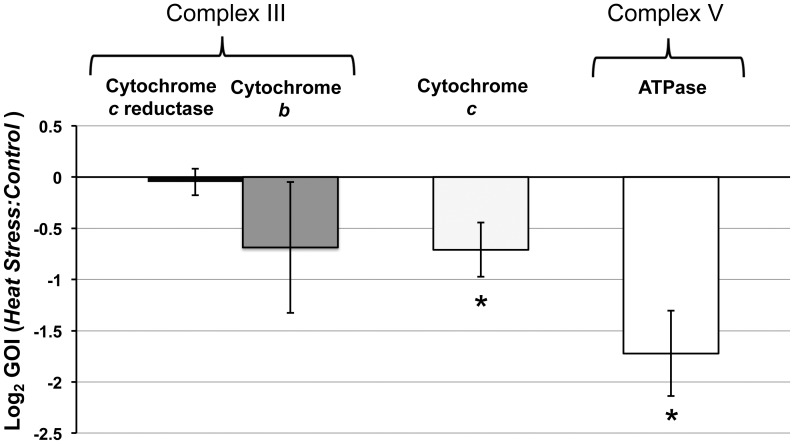
The ratio of Genes of Interest (GOI) associated with host mitochondrial electron transport and ATP synthesis with expression in thermal stress treated anemones versus controls.

To identify potential sites responsible for initiation of mitochondrial degradation in response to temperature stress, the study focussed upon the expression of genes associated with electron transport and ATP production. All primers were verified as host-specific using aposymbiotic anemone, freshly isolated dinoflagellates from the symbiosis and cultured clade B cDNA template controls. The housekeeping genes (HKGs) selected through GeNorm showed no significant change in expression between controls and heat-stressed anemones (S2: *t*_(6)_ = −132, P = 2.44; L19: *t*_(6)_ = 1, P = 0.354). The genes of interest (GOIs) were selected from complex III to V of the mitochondrial oxidative phosphorylation electron transport pathway with a focus around the shuttle protein cytochrome *c.* There was no significant difference in the expression of the cytochrome *c* reductase (*t*_6_ = 0.12_,_ P = 0.907) or cytochrome *b*(*t*_(6)_ = 1.08, P = 0.330) components of complex III between control and heat-stressed anemones (Figure 7). However, there was a significant reduction in expression of cytochrome *c* in heat-stressed anemones when compared to control (*t*_(6)_ = 3.59, P = 0.016), with a mean 2.19 fold reduction (log_2_ −0.702±0.2 S E) (Figure 7). In addition, there was also a significant reduction in ATP synthase expression in anemones treated with elevated temperature when compared to control (*W*_(6)_ = 52, P = 0.045) with a mean 3.84 fold reduction log_2_ −1.72±0.41 S E) (Figure 7). Therefore, the changes in expression of target genes associated with electron transport, ROS mediation and ATP production in response to thermal stress appeared to significantly change only at, and further downstream of cyt*c*.

## Discussion

Mitochondria are often referred to as the powerhouses or batteries of the cell due to their central role in providing energy in the form of ATP that supplies and controls cellular pathways necessary to cell survival or suicide [48]. The outcomes of this study demonstrate that changes in cnidarian host mitochondrial integrity, ultrastructure, and gene expression in response to thermal stress could be independent of the integrity of symbiotic algae. In addition, the study identified genes involved in functional electron transport, ATP synthesis and mitigation of ROS generation for which significant changes in level of expression could be pivotal to organelle degradation and cellular dysfunction. Observations using a combination of vital dyes, fluorophores and confocal microscopy, indicated visible differences in mitochondria of host cells from control compared to heat-treated anemones. The swollen and distended appearance of some host mitochondria subjected to thermal stress indicated the integrity was impaired; the membrane potential was disrupted resulting in an ionic imbalance and subsequent increased volume of the matrix (Figure 1B and C). Membrane disruption and swollen mitochondria are morphological indicators of the onset of cell death, occurring in both uncontrolled necrosis, and controlled apoptotic and autophagic pathways [26], [49]. In respect to this, the activation caspases within heat-treated host cells verified apoptotic cell death activity (Figure 1D). However, the findings of the confocal analysis alone only served as an indication that host mitochondrial integrity was susceptible to heat stress.

The detailed ultrastructural analysis of cells from the tissues of controls and heat-treated anemones corroborated the findings obtained by confocal microscopy analysis. Changes in host and symbiont organelles and cells were clearly evident in response to thermal stress. Further, while some symbionts appeared to be normal within heat-stressed anemones, the mitochondria within the respective host cells appeared to be either normal and/or at different stages of degradation within the same cell (Figure 3A). In addition, the observation that some adjacent host gastrodermal cells showed signs of detachment or late stage apoptosis also indicated a mitigation of damage response (Figure 3B). Symbiotic dinoflagellates undergoing *in situ* degradation along with corresponding degrading host cells were also identified. The low levels of mitochondrial and dinoflagellate degradation in controls (Figure 4 and 6) were likely to be as a result of natural organelle and symbiont population turnover. However, in some control anemone cells, the detection of degraded host mitochondria co-occurred with the presence of degraded dinoflagellates. This feature may be a host response to dysfunctional symbiont recognition and indicate that the host cell death pathways are activated through mitochondria to remove the dying symbiont. The highly significant changes in proportional distribution of mitochondrial and dinoflagellate degradation in heat-treated anemones (Figure: 4 and 5) occurred in all measured association scenarios (Figure 6). Most notable was that in heat-treated anemones the host cells had normal mitochondria with degraded dinoflagellates, and there was an increase in degraded mitochondria in host cells with symbionts with a normal appearance, indicating that, when subjected to thermal stress, host mitochondrial degradation is temperature-dependent and occurs independently of changes in the integrity of any symbiotic dinoflagellate. The disparity between these sets of observations suggests that no single unilateral chain of events characterises the cellular breakdown of the symbiosis within, and between each respective host cell and its symbiont during a response to thermal stress.

To identify potential initial sites of degradation within the host mitochondria as a response to thermal stress, the expression of a selection of key genes in the function of the electron transport chain and ATP was analysed by quantitative RT-PCR. Any potential changes in expression of the key genes could affect ATP content, the quantity and release of pro-apoptotic proteins such as cyt*c*, changes in mitochondrial membrane potential and integrity, solute and osmotic flux and cell death activation [50], [51]. The results of this analysis showed a small, but not significant reduction in cytochrome *c* reductase and cytochrome *b* of complex III within the host mitochondrial electron transport chain. However, there was a significant reduction in the expression of cyt*c* and ATP synthase (complex IV) downstream of complex III that could have important implications for the regulation of electron transport, ROS damage, ATP synthesis and controls over cell death activation, which are discussed further.

In this study, host anemone gastrodermal cell apoptosis was identified in thermally-stressed individuals by caspase activation and electron microscopy, and comparable to previous studies by Richier *et al.* [42], Dunn *et al.* [20], and again recently by Pernice *et al.* [32], Tchernov *et al.* [52] and Kvitt *et al.* [33]. The release of cyt*c* is one pivotal process in the onset of apoptosis and can be initiated as a result by overexpression of cyt*c*, which then reaches a maximal capacity within the intermembrane space of the mitochondria and a corresponding leaking into the cytosol through pores of the outer membrane [49]. However, the significant reduction of cyt*c* and apparent activation of cell death pathways in heat-treated anemones indicates that any potential release of cyt*c* and degradation of mitochondria could occur through alternative routes. This may precede or follow swelling and changes in mitochondrial membranes and homeostasis, which then leads to caspase activation and cell deletion [22], all of which were observed in heat-treated anemones.

**Figure 8:**
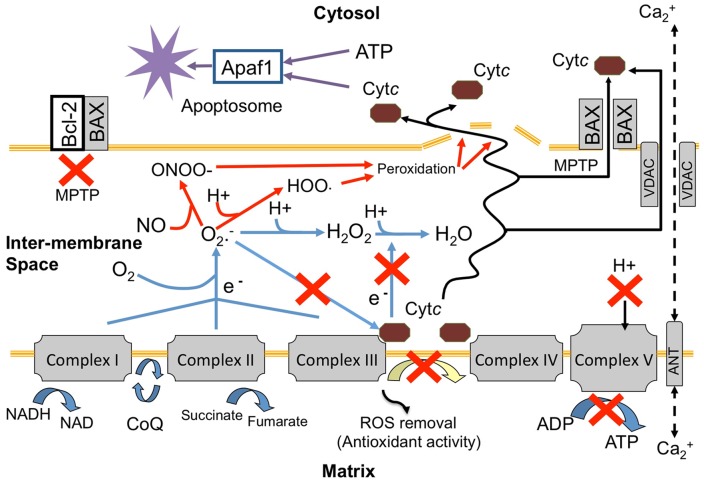
Schematic model of mitochondrial breakdown and representation of host cytochrome c (cyt*c*) interactions with electron transport, reactive oxygen species (ROS) and release mechanisms leading to cell death within hyperthermic stressed cnidarian hosts. Red X  =  where potential breakdown in pathway activity could occur as a result of reduced expression of cytochrome *c* and ATPase. Adapted from [58].

One potential interaction for the permeabilization of the outer mitochondria membrane and release of cyt*c* is the interaction of pro-apoptotic and anti-apoptotic Bcl-2 family members. In the present study, the morphological changes observed in heat-treated host mitochondria (Figures. 1B and C, 3A and 3B) may indicate activity of Bcl-2 to Bax expression as recently shown within the symbiotic coral, *Acropora millepora* in response to thermal stress recently shown by Pernice *et al.* [32], and a subsequent VDAC-ANT complex release of cyt*c*. [22]. The proportional ratio of pro-death signals such as Bax that initiate the membrane permeability transition pore (MPTP) can be out-competed by the actions of anti-apoptotic Bcl-2 in higher proportions that heterodimerize with pro-apoptotic signals to block MPTP formation (Figure 8) [53]. Alternatively, the ratio of pro to anti-apoptotic Bcl-2 family members may be involved in stimulating the opening of a voltage-dependent anion channel (VDAC), which can connect through to the matrix via an adenine nucleotide translocator protein complex (ANT), which cytosolic solutes can enter, resulting in mitochondrial swelling and outer membrane rupture as observed in this study (Figure 8) [22], [54], [55]. The VDAC-ANT complex may also be opened by changes in ROS, Ca^2+^ and pH [26]. In this respect, it is important to note that the anti-apoptotic protein Bcl-2 can also increase redox capacity and quench the effects of ROS and lipid peroxidation [56].

Mitochondria are known as the primary generators of ROS within animal cells including those of the cnidarians [35], but it is how the mitochondria deals with abnormal ROS generation that is key to its homeostasis [57]. It has previously been shown that even under normal circumstances, there is a level of electron leakage at complex I, II and III [58] that has the potential to generate ROS that, which if unchecked can cause membrane and protein damage through peroxidation. In this study, the expression of mitochondrial encoded cytochrome c reductase and cytochrome *b* of complex III did not significantly differ from controls indicating that these components were not a potential primary site of degradation. However, the significantly reduced level of gene expression downstream of complex III may indicate that complex III is a potential tipping point where electron transport disassembly takes place and a site for increased ROS production. The formation of ROS within the mitochondria intermembrane space can be quenched with the antioxidant role of cyt*c* [58]. Therefore the significant reduction in cyt*c* expression in response to thermal stress reported here could result in increased unchecked ROS generation and damage due to reduced antioxidant capacity and electron transfer between complex III and IV (Figure 8).

Although antioxidant enzymes such as MnSOD [13], [59], and catalase [60] have previously been shown to be present within symbiotic cnidarian tissues and play roles in cnidarian bleaching, the majority of mitochondrial antioxidant enzymes are in the matrix, and not within the intermembrane space, which is where the important antioxidant activity is undertaken by cyt*c* (Figure: 8). In different studies using vertebrate heart muscle preparations, the ROS generation in the form of O_2_-.and H_2_O_2_ has been shown to increase 7–8 fold with cyt*c* depletion, highlighting the important role of this shuttling protein [58]. Any reduced expression, and therefore antioxidant capacity, of cyt*c* could result in an increased potential for ROS interaction with secondary longer-lived signalling species, such as NO and protons in the intermembrane space, the formation of peroxynitrite and perhydroxyl radicals and therefore increased lipid peroxidation, membrane damage and cyt*c* release (Figure 8).

Mitochondria are also an important store for intracellular Ca^2+^, which is involved in apoptotic, necrotic and autophagic cell death processes [48]. Changes in Ca^2+^ regulation during hyperthermic stress- induced bleaching have been addressed previously in other studies [61], [62], [63] and were highlighted in a microarray study of bleaching in the coral *Monastrea faveolata* by Desalvo et al [64]. Components of the VDAC and MPTP participate in Ca^2+^ homeostasis and under cellular disruption, efflux of Ca^2+^ from the endoplasmic reticulum (ER) leading to mitochondrial swelling and release of cyt*c* [51]. The release of Ca^2+^ from the ER may be prevented by protective binding of Bcl-2 [51], corresponding to upregulation of Bcl-2 during bleaching shown by Pernice et al [32]. The features of mitochondrial degradation reported as outcomes of this study may also pertain to the known correlation between a reduction in ATP production and mitochondrial release of Ca^2+^and cyt*c* that in turn could affect controlled mitigation of damage through programmed cell death to uncontrolled necrosis (Figure 8) [25], [48], [50], [51].

An additional key point to consider is the potential influence upon cell damage mitigation and death processes of having broadly dispersed organelles around the periphery of the distended host symbiotic cell surrounding an endosymbiont. The increased distance and obstruction between host cell organelles may have consequences for intracellular communication and the translocation of compromised mitochondria to the site of apoptotic cell death communication with both the ER and nucleus. If a degraded organelle is essentially trapped by the stretched expansion of the host cell, this may result in independent organelle degradation and a failure of apoptotic controls leading to increased cell necrosis in symbiotic cells under increased levels of stress.

In conclusion, the cellular events occurring during the early hours of the onset of a hyperthermic stress, that is known to cause bleaching in the symbiotic anemone model system, were characterized, in the present study, by the loss of mitochondrial integrity together with a disruption and down-regulation of genes associated with electron transport and ATP production downstream of complex III. In contrast to control conditions, the loss of host mitochondrial integrity appeared to be driven by temperature increase independently of the symbiotic dinoflagellate condition, suggesting that the host mitochondria in some cases may be the initial point of symbiosis disruption, which reciprocates the findings of Dunn *et al.* [45] and later by Ainsworth *et al.* [65], corresponding to the release of photosynthetically competent dinoflagellates during bleaching [66]. The conclusions of this study do not rule out the existence of ROS generation, organelle and cellular dysfunction and death within the dinoflagellates during the breakdown of the symbiosis. However, our results clearly show that symbiont degradation does not have to be the primary event during response of the breakdown of the cnidarian-dinoflagellate endosymbiosis. In fact, the identified changes in electron transport and ATP downregulation during host mitochondrial disruption have potential complex ramifications for host cell homeostasis during thermal stress. Further studies are clearly needed to investigate potential increases in mitochondrial ROS, intracellular Ca^2+^ and cyt*c* release, and how mitochondrial degradation correlates to tissue metabolic turnover. A further conclusion from this study was that not all mitochondria responded simultaneously or uniformly to the initial effects of the hyperthermic stress and implies that continued intracellular ATP production is available to permit apoptosis/autophagic completion or to mitigate necrotic outcome of mitochondrial perturbation.

## Methods

### Culture Conditions

The cultures of the symbiotic sea anemone, *Aiptasia pulchella* were maintained under a 12 h light (350–400 µmol m^−2^/s^−1^from a metal halide unit)/12 h dark regime for over a year. Temperature was regulated at 25–26°C and cultures were fed once a week with frozen *Artemia* spp. nauplii. Individual anemones were selected from the main culture and placed in a separate tank under culture conditions for a week prior to an increase in temperature over 18 h to 32–33°C, which was maintained for a period of 24 h. In previous studies, similar doses of thermal stress have been shown to elicit a bleaching response due to a loss of dinoflagellate symbionts [20]. However, for the purpose of this study, repeated analysis of bleaching (loss of pigment and symbiont population) was not undertaken. Anemones were removed after 24 h from both control and thermal stress treatment tanks simultaneously for processing.

### Tissue Sectioning, Vital Dyes, Fluoroprobes and Laser Confocal Microscopy

The embedding and sectioning of whole anemones was adapted from Dunn *et al.* [20]. Sample anemones were removed from treatment and controls cultures then placed in 0.36 M MgCl_2_ in seawater for 30 min prior to being embedded in TBS tissue freezing medium (Triangle Biomedical Sciences), and stored at −80°C. Tissue sections (20–25 µm) were produced using a Leica CM3050 S cryostat microtome**,** mounted within a PAP (Daido Sangyo Co, Ltd) pen-restricted area of poly-L-lysine coated slides (Mezel-Glaser), and returned to −80°C. Slides were later removed from the −80°C, allowed to briefly thaw, rinsed in phosphate buffered saline (PBS: 2 mM, NaH_2_PO4, 7.7 mM, Na_2_HPO_4_, 0.14 M NaCl in H_2_O, pH 7.0) for 2 min at room temperature. Sections were then treated with a stain mixture consisting of Hoechst 33342 DNA stain (Molecular Probes- Invitrogen) (200 µg ml^−1^) to visualize host nuclei and Mitotracker-Red Fm dye (Molecular Probes- Invitrogen) (FC 500 nM) to stain all mitochondria and kept in the dark at room temperature (25°C) for 1 h. Following incubation, samples were rinsed twice in PBS to remove excess dye. Excess PBS was removed by tapping the slides, which were then mounted using AR1 glycerol PBS mountant (Citifluor). Coverslips were sealed and the slides viewed under a Zeiss LSM 510 metahead confocal microscope. Samples were scanned with excitations of 405 nm and 543 nm to visualize the emission spectra of 461 nm for Hoechst 33342 and 644 nm for Mitotracker dyes respectively. As a positive control for caspase activation, individual anemones were separately incubated in 0.5% solution of colchicine in sea-water, a known inducer of apoptosis [67] for 24 hrs and then processed and sectioned as above.

### Electron Microscopy

As part of this study, the techniques of high-pressure cryofixation and freeze substitution were adapted for use with the cnidarian-dinoflagellate tissues and cells as this enables a greater ultrastructural resolution of cells and membranes, which represents a greater true representation of cells *in situ* than pre-chemical fixation techniques [68], [69]. Whole anemones from control and thermal stress treatments (n = 3 replicates per treatment) were anesthetized in 0.36 M MgCl_2_ as described above. Whole animals were removed from the anesthetic, and along with immediately excised tentacles, placed in interlocking brass hats (moulds) or aluminium planchettes and cryoimmobilised in cryoprotectant (20% BSA in artificial seawater) using a Bal-Tec HPM 010 high pressure freezer. Samples were then transferred under liquid nitrogen to an automated freeze substitution machine (AFS2, Leica) where they were freeze-substituted in a solution of 1% OsO_4_, 0.5% uranyl acetate and 5% water in acetone for 48 h at −90°C before being brought to room temperature. Samples were washed in acetone and infiltrated with Epon resin (Epon 812, ProSciTech) using a Pelco Biowave microwave. Sections were cut at 70 nm on a Leica UC6 ultramicrotome and viewed at 80 kV in a Jeol 1011 transmission electron microscope. Images were captured with a Morada digital camera using AnalySIS software (Olympus). Electron micrographs were examined to assess both host mitochondrial and dinoflagellate integrity within individual cells for each one of the three animals from the hyperthermic treatment and control. In order to assess the required large amount of organelles randomly and throughout the tissues to indicate any changes in integrity as a response to heat, the additional large number of serial sections required to accurately determine the size of each organelle was not undertaken. Instead, the morphology of the respective membranes, including cristae and dinoflagellate thylakoids were used as indicators for assessment of integrity. The integrity of gastrodermal host mitochondria was determined upon morphology of the organelle cristae and double membrane. Normal cristae and membranes were clearly defined and intact as opposed to degraded cristae and membranes, which were ragged, broken, fragmented or distorted and separated. In addition, the integrity of the symbiotic dinoflagellates was based upon the integrity of the chloroplast thylakoid membrane and that separation would indicate primary degradation, in accordance with Dunn *et al.* [2] and Tchernov *et al.* [44]. Where anemone gastrodermal cells did not contain dinoflagellates, this did not mean that a dinoflagellate was not present, but scored as not observed. Verification of symbiont presence with host cells could have only been possible using serial sections and enhanced replication for statistical analysis, but as with the organelle size measurement, was beyond the scope of this study. The proportion of degraded mitochondria and dinoflagellates were established for control and heat-treated animals. The mitochondrial counts for each animal were pooled for the hyperthermic stress treatment (n = 562) and control animals (n = 413) and the proportional distribution of degraded and normal mitochondria and dinoflagellates established from individual cell analyses.

### Caspase Activity Assay

The fluoroprobe rhodamine 110 aspartic acid caspase substrate (Molecular Probes) was used to detect the activation of caspase proteases and associated apoptotic cell death. Activation was indicated by cleavage of rhodamine 110 substrate resulting in a fluorophore signal detectable with confocal microscopy. To detect and assess changes in caspase and therefore apoptosis activation within host tissues and potentially symbionts, tentacles were excised from the treated and control anemones that were incubated within 0.36 M MgCl_2_ in seawater for 30 min. The excised tentacles were then placed in dark 1.5 ml microfuge tubes containing 200 µM final concentration rhodamine 110 caspase substrate (dissolved in 0.2% DMSO) in seawater for 1 h. Hoechst 33342 DNA stain (200 mg ml^−1^) was also added to visualize host nuclei. Samples were briefly rinsed in PBS prior to mounting with AR1 mountant (Citifluor) and coverslips as above. Tissue sections were viewed under the laser confocal microscope with excitation wavelengths of both 405 nm for the Hoechst 33342, and 543 nm for the rhodamine 110 with an emission of 521 nm for the caspase activity. A total of about 12 sections obtained from the 3 replicates per treatment per time point were visually examined for fluorophore signal.

### Quantitative RT-PCR (qRT-PCR)

The present study conforms to the Minimum Information for Publication of Quantitative Real-Time PCR guidelines [70]. In this section, we report the minimal essential information sensu [70] required to allow reliable interpretation of the corresponding qRT-PCR results.

### RNA Extraction for Aiptasia Pulchella

Whole anemones were immediately snap frozen in liquid nitrogen and stored at −80°C. Samples were removed and individually ground in liquid nitrogen to a fine powder using a sterile pestle and mortar. The frozen powdered sample was placed in a sterile 1.5 ml tube prior to extraction. Total RNA was then extracted from the aqueous phase with RNeasy kit (Invitrogen) according to manufacturer’s instructions and checked for quantity and integrity using an Agilent 2100 bioanalyzer. A total of 2 ng of high-quality total RNA (integrity number >7) for each sample was used for cDNA synthesis using the Quantitec reverse transcriptase kit™ (Qiagen) including a DNAse step to remove contaminating genomic DNA in accordance with manufacturer’s instructions.

### Selection of Genes of Interest (GOI) and Primer Design

The full open reading frame of 402bp (133 amino acids) (Accession No: HE805692) for the protein cyt*c* for *Aiptasia pallida* was initially established using PCR amplification with a series of degenerate primers, triplicate clone sequences of the final reads and cDNA phage library template. The complementary sequence for *A. pulchella* cyt*c* was mapped against the *A. pallida* sequence and found to be identical aside from a synonymous mutation from adenine to thymine at position 120 within a glycine residue. The ORF for *Aiptasia* spp. cyt*c* blastx results aligned closest to complementary sequences of the cnidarian, *Nematostella vectensis* predicted protein (e value  = 1e^−49^; Identity 92/102 (90%) and the full cyt*c* ORF of *Pectinaria gouldii* (e = 2e^−49^; Identity 91/108 (84%). A confirmed translation protein sequence (ExPASy) pblast returned cyt*c* domains (e = 1.6e^−40^). The qRT-PCR primers designed from the open reading frame sequence are shown in Table 1).

**Table 1:** Housekeeping genes and genes of interest investigated in *Aiptasia pulchella* by using qRT-PCR.

Gene Name	Accession Number/Reference	5′–3′ Primer Sequence	3′–5′ Primer Sequence	Size (BP)
*Aiptasia pallida* Cytochrome *c*reductase subunit 2	GH573837.1	GAGCAACAGCCCCAAGCA	CTGCGCTTTTGCCTGGTAAA	90
ATP synthase mitochondrialsubunit 6	GB AAC04634.1 (*M. senile*) and GB| ABG02353.1 (*Nematostella sp.*)	GGTGTAACTTTAGGTGGGCTT	ATTTGCGGCGAGACGGAC	159
Aiptasia spp. Cytochrome *c*	HE805692	AGGAATCACATGGGGTGGGGTGAAG	ATAAGGTCTGCACGCTCGTT	120
*Aiptasia pulchella.* Cytochrome *b*	S75445: *M. senile* DQ643835.1 (*Nematostella sp.)*	CATGCCAATGGAGCCTCC	CCAAAAGGACATTTGTCCCCA	177
Ribosomal protein S2 (HKG)	AiptasiaBase Contig1425 Pernice*et al.* 2010	TAGAGCTGGTCAGCGAACAA	AACCACGCCTAACAGGAATG	173
Ribosomal protein L19 (HKG)	AiptasiaBase Contig541 and CCAS691.g1	TGCAAACGCTAACTCACGTC	GCATACGAGCATTGGCAGTA	158

For all other genes of interest (GOI), the design of primers was based either on sequences previously known or on sequences isolated and characterized in this study. In-depth analysis of an annotated database generated by sequencing of *Aiptasia pallida* (AiptasiaBase: http://aiptasia.cs.vassar.edu/AiptasiaBase/index.php [71]) revealed a transcript-encoding protein with high similarities to cytochrome *c* reductase (Accession: GH573837.1). The mitochondrial ATP synthase and cytochrome *b* sequences of *Aiptasia* sp. were unavailable prior to this study. An initial set of PCR primers was therefore designed, based on a consensus of ATP synthase subunit 6 sequences and cytochrome *b* reductase subunit 2 sequences from the anemones *Metridium senile* and *Nematostella* sp. An initial partial sequence for ATP synthase subunit 6 of 346bp was amplified using the forward primer indicated in Table 1 and a reverse primer (3′- GATGGTATCAGCTAAATAAATAG-5′). This sequence aligned with both *M. senile* and *Nematostella* sp. ATP synthase subunit 6 sequences with xblast E values of 6E^−46^ and 7E^−42^ and identities of 116/116 (100%) and 105/116 (91%) respectively. The partial sequence for cytochrome *b* of 333bp was obtained using a forward primer 3′- ATGTGGAACTTCGGTTCTTTA and the reverse primer indicated in Table 1. This sequence aligned again with *M. senile* and *Nematostella sp*. cytochrome *b* sequences with xblast E values of 1e^−57^ and 4e^−56^ and identities of 102/111 (92%) and 100/111 (90%) respectively. The partial sequences obtained in this study for ATP synthase subunit 6 and cytochrome *b* were then used as templates to design sequence-specific primers for qRT-PCR (Table. 1) using the software, Primer3 [72], (Source code available at http://fokker.wi.mit.edu/primer3/). Primer specificity to host genes was tested and confirmed by using cDNA from aposymbiotic *A. pulchella* and sequence identity.

### Selection and Normalisation of Housekeeping Genes (HKG)

The initial pool of potential reference genes used in this study was obtained through previous studies [73], [74](see Table. 1). In order to select the best HKGs for the experimental conditions, expression stability was analysed using GeNorm software [75]; (http://allserv.ugent.be/jvdesomp/genorm/index.html). Under our different experimental conditions (control and heat-stress treatments), the most stable expression was revealed for genes coding for ribosomal proteins L19 and S2 (M value = 0.284, Figure S1A). A minimum of 2 reference genes (L19 and S2) was recommended for accurate normalization of gene expression in the samples incubated with heat stress (V2/3 = 0.127, Figure S1B).

### Differential Gene Expression

Quantitative RT-PCR assays were set up using an Eppendorf epMotion 5075 Robotics System (Eppendorf) in accordance with Pernice *et al.* [32]. Efficiency of target amplification was optimised prior to running samples for each primer pairs. Constant CT values for each primer set were observed at a final concentration of 500 nM for each of the primer pairs and we verified that the dissociation curve yielded a single peak indicating specific amplification of the target amplicon. Aliquots of cDNA from each sample served as templates for qRT-PCR using SYBR Green PCR Master Mix (Applied Biosystems) on an Applied Biosystems 7900 Real-Time PCR System. Amplification of 10 µl reactions of cDNA (normalised to RNA content) from control and hyperthermic stress treated samples, and 500 nM of each specific primers, were placed in 384-well optical plate (Perkin Elmer/Applied Biosystems Divisions) under the following conditions: incubation at 50°C for 2 min, then at 95°C for 10 min, 45 cycles of 95°C for 15 sec and 60°C for 1 min. Real-time PCR efficiency for each gene and each treatment were determined from a cDNA dilution gradient of 90, 30, 10, 3 and 1 ng and linear regression model [76]. The corresponding real-time PCR efficiencies were calculated according to the equation described by Radonic *et al.* [77]:
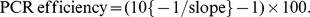



All qRT-PCR displayed efficiencies between 91% and 104% (r^2^ of calibration curve >0.99). A no template control as well as a no reverse transcription control was generated for each gene and each treatment to ensure that the cDNA samples were free of DNA contamination.

### Data Acquisition

Data from qRT-PCR were analysed using the Sequence Detection Software (SDS 2.2). Expression levels were determined as the number of cycles needed for the amplification to reach a fixed threshold in the exponential phase of PCR reaction [78]. The cycle threshold (CT) was set at 0.05 for all genes, and corresponding CT values were transformed into quantities using PCR efficiency according to Vandesompele *et al*. [75] in order to use GeNorm software (http://allserv.ugent.be/jvdesomp/genorm/index.html). For each individual GOI, the expression level was measured in accordance with Pernice *et al.* [32]. HKGs for this experiment were analysed using GeNorm software according to Vandesompele *et al.* [75]. The ribosomal proteins L19 and S2 were selected as being the most stable HKG. Real-time PCR efficiency values for each sample triplicate were determined using a standard cDNA dilution gradient [32] as described above and corrected using LinReg software [79].

### Statistics

The total counts of host mitochondria and dinoflagellates displaying normal or degraded ultrastructural morphology within anemones from the heat treatment and controls were normalized to the lowest counts (controls) for statistical comparison. The distribution of degraded and normal mitochondria and dinoflagellates within control and heat-treated anemones were tested using the normalized counts, contingency tables and multiple χ^2^ tests. The total counts for control and heat-treated anemones were also pooled to assess proportional distribution and were separated into 6 different association categories (Figure 6). The level of gene expression of each individual GOI was normalized to the level of expression of L19 and S2 using the Normalization Factor (NF) given by GeNorm. Normalized expression values of GOI were then compared between individuals from heat stress and control treatments. Data were tested for normality, homogeneity of variance and heteroscedasticity and transformed accordingly using a log_10_ transformation. When the data deviated from normality and/or were not homogeneous, then non-parametric tests (Mann-Whitney) were applied instead of parametric tests (paired *t*-test) used for normally distributed, homogenous data using Minitab™ statistical software v16. Mean values are reported for 6 anemones ± standard error (SE). Results were considered significant at the P<0.05.The GOI differential expression between control and treatment is presented on a log^2^ scale for similar visualisation of up and down gene regulation.

## Supporting Information

Figure S1 (A) Average expression stability values of candidate House Keeping Genes (HKG) determined by GeNorm analysis under different experimental conditions. (B) Determination of the optimal number of HKG required for accurate normalisation by GeNorm analysis.
(DOCX)
