# Supplementary figures and images for: Thermal Stress Promotes Host Mitochondrial Degradation in Symbiotic Cnidarians: Are the Batteries of the Reef Going to Run Out?
Source: PLoS One. 2012 Jul 16;7(7):e39024. doi: 10.1371/journal.pone.0039024 (PMC3398039; doi:10.1371/journal.pone.0039024)

**Supplementary Figures:**


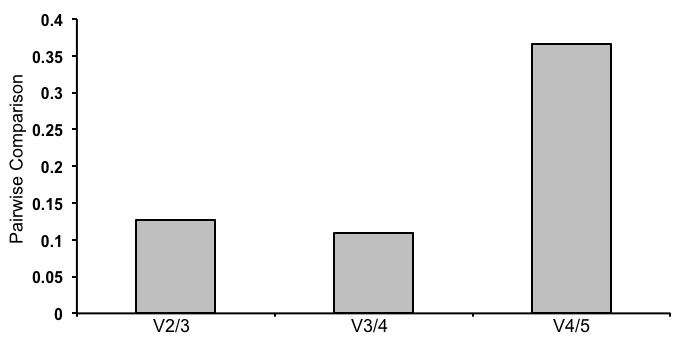


Figure: S1A


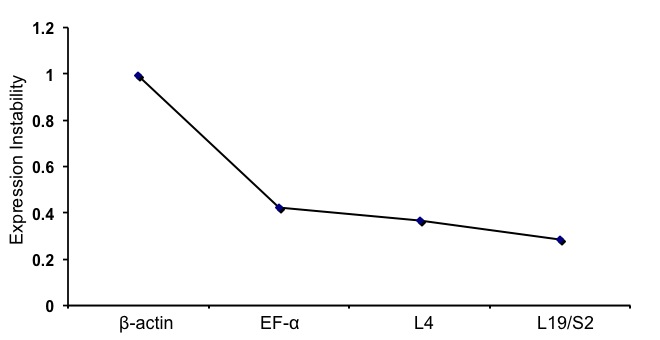


Figure: S1B

Supplement: Figure S1 — (A) Average expression stability values of candidate House Keeping Genes (HKG) determined by GeNorm analysis under different experimental conditions. (B) Determination of the optimal number of HKG required for accurate normalisation by GeNorm analysis. (DOCX) [file pone.0039024.s001.docx]
